# Iris Depigmentation: An Unusual Complication of Intralesional Corticosteroid Injection for Capillary Hemangioma

**DOI:** 10.4103/0974-9233.61226

**Published:** 2010

**Authors:** Huda Al-Mahdi

**Affiliations:** Departement of Opthalmology, Ophthalmology Section, Hamad Medical Corporation, PO Box 3050, Doha-State Qatar

**Keywords:** Capillary Hemangioma, Iris Depigmentation, Steroid Injection

## Abstract

Intralesional injection of corticosteroids has been used successfully in the treatment of adenexal hemangiomas, with advantages of easy administration, rapid action, repeatability, efficacy and safety. We report a case of an eight-month-old female infant who underwent intralesional corticosteroid injection for capillary hemangioma that had resulted in amblyopia of her left eye from ptosis. Two weeks after the injection, the hemangioma showed some regression but at that point iris depigmentation of the affected eye was noted. The iris depigmentation remained unchanged during her follow-up visit with significant regression of the hemangioma, associated ptosis and astigmatism.

## INTRODUCTION

Capillary hemangioma is a benign vascular tumor of infancy that commonly involves the eyelids and orbit. The rate of incidence of these tumors among newborns is 1–2%.^1^ They usually grow rapidly during the first 6–12 months of life followed by gradual, spontaneous, involution starting at about two years of age. Complete resolution occurs in 40% by the age of four years and 70% by seven years. Because of the high incidence of associated amblyopia, especially with large adnexal hemangiomas, several treatment modalities have been used, including intralesional steroid injection.^2^ Other modalities include systemic steroids, interferon, surgical excision, cryotherapy and radiotherapy.^6^ Kushner reported successful treatment of infantile adnexal hemangioma using intralesional steroid injections although complications such as fat atrophy, eyelid necrosis^3^ and central retinal artery occlusion have been reported.^4^ However, it is still considered as a safe, and effective method of treatment. We report a patient who developed unusual left iris depigmentation following intralesional corticosteroid injection for a capillary hemangioma of the left upper eyelid that was causing amblyopia.

## CASE REPORT

A three-month-old female infant, born via normal delivery, was examined for a mass involving the left upper eyelid. Her parents stated that her eyelids appeared normal at birth but at two weeks of age a mass appeared in the infants left upper eyelid which rapidly enlarged over a month.

**Figure 1:**
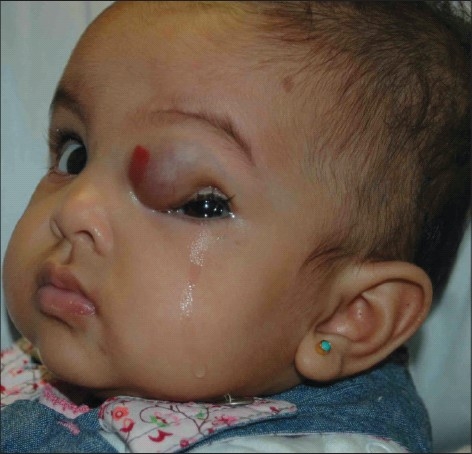
A three-month old female infant with left upper lid hemangioma causing marked ptosis

On examination, the fixation pattern was central, steady and maintained in the right eye, and she was able to follow light through a slit-like opening of the left palpebral fissure. There was a 3.5 × 3.00 cm elevated mass with an overlying red spot, involving the medial two third of the left upper eyelid, that caused a marked ptosis in that eyelid [Figure 1]. Ocular motility was full in both eyes with limited elevation in the left eye. There was no afferent pupillary defect. The color of the iris was dark brown in both eyes, and the fundus was normal in both eyes. Cycloplegic refraction of the right eye was +1.50 + 1.50 × 90° and for the left eye was + 5.00 –8.00 × 40°.

Computed axial tomography with contrast showed a large hyperdense extraconal lesion located at the superomedial aspect of the left orbit causing inferolateral displacement of the globe. The rest of the examination was within normal limits and a diagnosis of infantile capillary hemangioma was made. Because of the high risk of developing amblyopia from visual deprivation and ptosis, the infant was started on a five-week course of tapering oral prednisone starting at a dose of 5 mg, twice a day for two weeks while being monitored by a pediatrician. At the end of treatment, there was some regression in the size of the tumor that now measured 2.50 × 2.00 cm. The cycloplegic refraction was now +3.00 D Sph –6.50 D Cyl × 40°.

**Figure 2:**
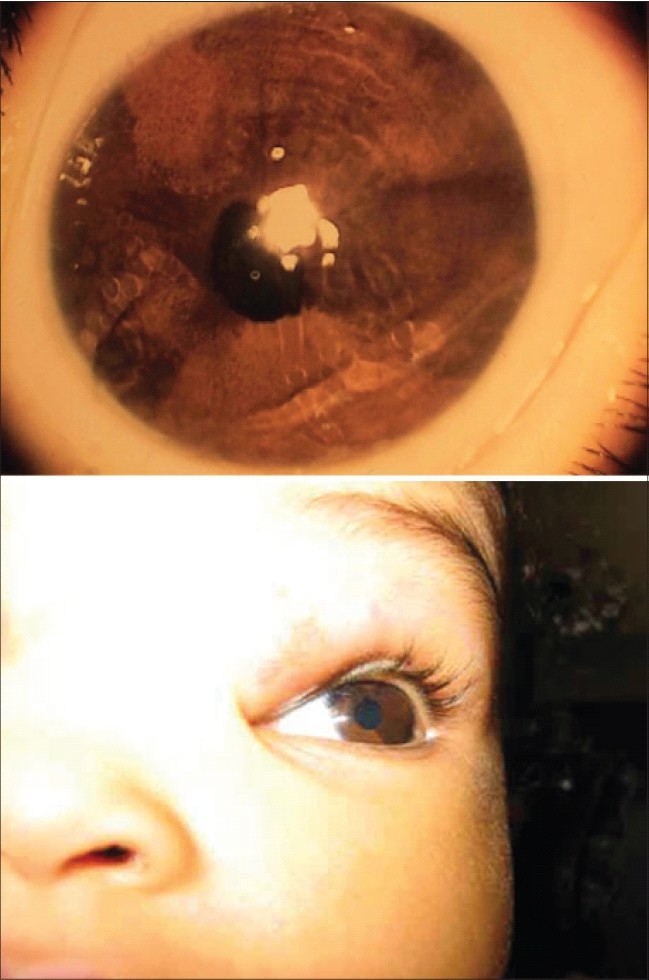
Left eye iris hypopigmentation, three weeks following intralesional corticosteroid injection

**Figure 3:**
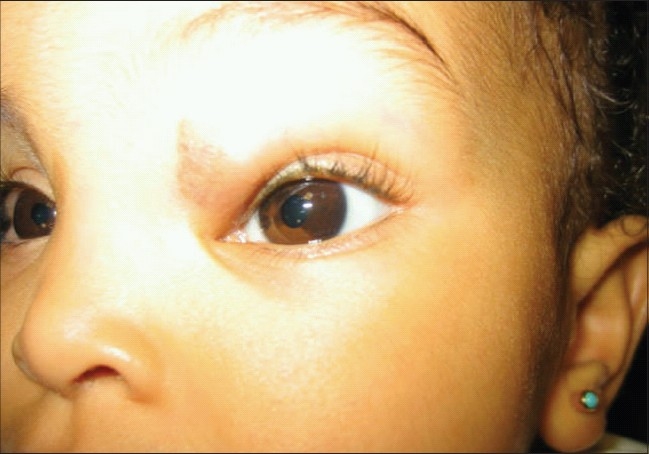
Hypopigmented area of left iris remains the same in the follow-up visit

After several subsequent visits, there was no further regression in tumor size and astigmatic error remained. Therefore a mixture of 40 mg of triamcinolone acetonide and 8 mg of dexamethasone sodium phosphate was administered within the lesion under general anesthesia. The injection was given using a 27-gauge needle on a 1-ml tuberculin syringe at multiple injection sites throughout the lesion. A patch was applied over the treated eye and she was discharged. The immediate postoperative course was uneventful. At 3-4 weeks after treatment the patient followed light with both eyes, with nor mal fixation with marked reduction in the size of the tumor to 1.50 × 1.00 cm. On examination of the anterior segment, an area of hypopigmentation involving the left iris tissue was noted [Figure 2]. The pupil was normally reactive, funduscopy was anormal and cyclopelgic refraction was plano –6.00 axis 40°. Follow-up visits showed that the area of hypopigmentation remained unchanged [Figure 3]. On the last visit which was one year after treatment her visual acuity was 6/24 in her left eye, using Teller acuity measurements. Ultrasound bio-microscopy of the left iris was unremarkable. Imaging was also repeated which showed a regressed hemangioma with normal brain.

## DISCUSSION

Capillary hemangiomas are benign tumors composed of proliferating endothelial cells and anastomosing blood filled channels.^5^ As they usually follow a pattern of spontaneous gradual regression over the next few years, treatment is not required unless the hemangioma is large enough to cause occlusive or refractive amblyopia. Several methods of treatment have been described but each has their own merits and risks. Intralesional corticosteroid injection was introduced by Kushner in 1979 and is considered as the treatment of choice.^2^ Although side effects have been reported with this treatment, the incidence is low and has involved eyelid depigmentation,^6^ local subcutaneous fat atrophy,^7^ central retinal artery occlusion^8^ and eye lid necrosis.^3^ The corticosteroids are believed to increase blood vessel sensitivity to circulating catecholamine causing vasoconstriction, not due to their anti-inflammatory effect.^9^

The mechanism of iris depigmentation following intralesional corticosteroid injectionin our case remains unclear but steroids could possibly have been mediated by its effect in melanocytes. Steroids may reduce the number, or activity, of melanocytes.^10^ Also, glucocorticosteroid receptors are found in many cells including melanocytes in skin and iris.[6] Callen^11^ noted that intralesional corticosteroid injections in dermatological applications might cause atrophy, telangiectasia, and alteration in skin pigmentation that depended on the strength of preparation, the quantity injected and the area of body being treated. Corticosteroids appear to inhibit melanin synthesis and cause melanocytes to assume an effete appearance. Skin hypopigmentation has been documented in many cases following intralesional steroid injection, and in most such cases it resolves over weeks.^11^ However, there have been reports of unresolved hypopigmentation in a few cases.[10]

In summary this report highlights iris depigmentation as an unsual side effect of intralesional steroid therapy that has not been previously reported. Further studies are needed to determine the exact etiology of this complication.
